# Spermatocytic seminoma at the national institute of oncology in Morocco

**DOI:** 10.1186/1756-0500-4-393

**Published:** 2011-10-10

**Authors:** Ghizlane G Raiss, Marwane M Benatiya Andaloussi, Soundouss S Raissouni, Hind H Mrabti, Hassan H Errihani

**Affiliations:** 1 Medical oncology Department, National Institute of Oncology, Rabat, Morocco; 2 Department of Urology, Ibn Sina Hospital, Rabat, Morocco

## 

Following the publication of our article [1], we realised that we did not give correct attribution to our previous publication [2]:

Andaloussi M, Rais G, Raissouni S, Barki A, El sayegh, Iken A, Nouini Y, Lachkar A, Benslimane L, Mrabti H, Errihani H, Faik M. Seminome Spermatocytaire: a Propos d'un Cas et Revue de La Litterature Spermatocytic Seminoma. Afr. J. Urol. 2011 17 (1): 18-23.

We used material from the above publication: Figures 1 and 2 have been copied, and we have taken material from Table 2.

We apologise for any inconvenience this may have caused.
